# The Brain-Nose Interface: A Potential Cerebrospinal Fluid Clearance Site in Humans

**DOI:** 10.3389/fphys.2021.769948

**Published:** 2022-01-04

**Authors:** Neel H. Mehta, Jonah Sherbansky, Angela R. Kamer, Roxana O. Carare, Tracy Butler, Henry Rusinek, Gloria C. Chiang, Yi Li, Sara Strauss, L. A. Saint-Louis, Neil D. Theise, Richard A. Suss, Kaj Blennow, Michael Kaplitt, Mony J. de Leon

**Affiliations:** ^1^Undergraduate Department of Biology, Cornell University, Ithaca, NY, United States; ^2^Pelham High School, Pelham, NY, United States; ^3^Department of Periodontology and Implant Dentistry, NYU College of Dentistry, New York, NY, United States; ^4^Department of Medicine, University of Southampton, Southampton, United Kingdom; ^5^Department of Radiology, Brain Health Imaging Institute, Weill Cornell Medicine, New York, NY, United States; ^6^Department of Radiology, NYU Langone Health, New York, NY, United States; ^7^Department of Radiology, Weill Cornell Medicine, New York, NY, United States; ^8^Department of Pathology, NYU Grossman School of Medicine, New York, NY, United States; ^9^Division of Neuroradiology, Department of Radiology, University of Texas Southwestern Medical Center, Dallas, TX, United States; ^10^Clinical Neurochemistry Lab, Inst. of Neuroscience and Physiology, University of Gothenburg, Sahlgrenska University Hospital, Göteborg, Sweden; ^11^Laboratory of Molecular Neurosurgery, Department of Neurological Surgery, Weill Cornell Medical College, New York, NY, United States

**Keywords:** CSF, neuroimaging, cribriform plate, Alzheimer’s disease, neurodegeneration

## Abstract

The human brain functions at the center of a network of systems aimed at providing a structural and immunological layer of protection. The cerebrospinal fluid (CSF) maintains a physiological homeostasis that is of paramount importance to proper neurological activity. CSF is largely produced in the choroid plexus where it is continuous with the brain extracellular fluid and circulates through the ventricles. CSF movement through the central nervous system has been extensively explored. Across numerous animal species, the involvement of various drainage pathways in CSF, including arachnoid granulations, cranial nerves, perivascular pathways, and meningeal lymphatics, has been studied. Among these, there is a proposed CSF clearance route spanning the olfactory nerve and exiting the brain at the cribriform plate and entering lymphatics. While this pathway has been demonstrated in multiple animal species, evidence of a similar CSF egress mechanism involving the nasal cavity in humans remains poorly consolidated. This review will synthesize contemporary evidence surrounding CSF clearance at the nose-brain interface, examining across species this anatomical pathway, and its possible significance to human neurodegenerative disease. Our discussion of a bidirectional nasal pathway includes examination of the immune surveillance in the olfactory region protecting the brain. Overall, we expect that an expanded discussion of the brain-nose pathway and interactions with the environment will contribute to an improved understanding of neurodegenerative and infectious diseases, and potentially to novel prevention and treatment considerations.

## Introduction

The highly coordinated function of the human brain in regulating nearly all bodily functions entails a fundamental need to protect its anatomical and functional homeostasis. The nose-brain interface, modulating a critical exchange between the nervous system and the environment, plays a role in immune function and fluid clearance. Serving as a primary physical boundary against the ready invasion of the central nervous system (CNS) by pathogenic organisms, the nasal region mediates a physiological safeguard of the brain. Alongside its regulation of environmental microbes, the nose-brain connection also functions in Cerebrospinal Fluid (CSF) circulatory pathways as demonstrated across numerous species. CSF flow, widely accepted to be driven by arterial pulsations that provide a pumping force, facilitates the removal of metabolic byproducts, toxic macromolecules, and other important substances (Mestre et al., 2018). Its clearance maintains a homeostasis in the brain that prevents the toxic accumulation of compounds and allows for the brain to function under normal physiological conditions (Rennels et al., 1985; Ghersi-Egea et al., 1996; Tarasoff-Conway et al., 2015; Bakker et al., 2016; Benveniste et al., 2017). Clearance mechanisms involving cranial nerves, perivascular compartments, and lymphatic drainage, supplemented with a convective influx/glymphatic exchange between cerebral interstitial fluid (ISF) and CSF, ultimately regulate the dynamics of biomarkers.

Among these clearance mechanisms, the scientific documentation of an olfactory pathway dates back to the 19th century and marks one of the earliest described CSF efflux avenues (Schwalbe, 1869). Considerable experimentation in multiple animal species demonstrates the engagement of an olfactory drainage route of CSF through the cribriform plate into the lymphatic circulation of the nasal mucosa and epithelia. Despite the compelling evidence for the clearance of CSF *via* the nasal-nervous pathway in many mammals, the anatomy and physiological significance of this pathway in humans remain largely unclear. This review, aimed at directly examining the importance of *in-vivo* and post-mortem human studies, explores the potential physiological relevance of the CSF clearance pathway mediated by the nose-brain interface.

## Csf Production and Driving Forces

Cerebrospinal Fluid movement through the CNS begins largely with its production at a system of epithelial cells in the choroid plexus of the lateral, third, and fourth ventricles (Sakka et al., 2011). CSF is also produced *via* filtration through the capillary walls into the extracellular space of the surrounding brain tissue (Buishas et al., 2014). CSF in the lateral ventricle passes through the interventricular foramen of Monro into the third ventricle, flows into the fourth ventricle *via* the cerebral aqueduct, and then exits the foramina of Magendie and Luschka to circulate in the subarachnoid space around the brain and the spinal cord (Tumani et al., 2017; Khasawneh et al., 2018). The movement of CSF through nervous tissue culminating with clearance from the CNS is driven by a variety of factors. Recent insights into the mechanics of CSF flow suggest arterial pulsations play a role in promoting CSF movement in the brain (Iliff et al., 2013; Kedarasetti et al., 2020). Additionally, the established low resistance of flow pathways coupled with decreasing osmotic pressure gradients within the brain help facilitate the circulation of CSF. Healthy amounts of activity and rest may also play a role in regulating CSF clearance as does the restorative function of sleep. In particular, during slow wave sleep, the activation of an astrocyte modulated aquaporin system may allow for up to 60% enhancement of CSF clearance (Xie et al., 2013; Eugene and Masiak, 2015; Reddy and van der Werf, 2020). Ultimately, these factors combine to regulate the movement of CSF as it circulates through the brain following primary production at the choroid plexus of the ventricles.

## Glymphatic and Intramural Periarterial Drainage

Recent research shows that CSF can enter the brain parenchyma along periarterial compartments (Rennels et al., 1985; Iliff et al., 2012; Albargothy et al., 2018). Interstitial spaces in the adventitia around arteries and veins have been shown to be continuous across tissue planes and organ boundaries in other locations (e.g., skin and colon) and continuous with connective tissues of the organs served by those vessels (Benias et al., 2018; Cenaj et al., 2021); cerebral extracellular spaces connect with the basement membranes of the cerebral and leptomeningeal vasculature continuing to the basement membranes of extracranial vessels. Such spaces are, in part, likely to be the anatomical substrate of the “paravascular” and “perivascular” routes of flow. The “paravascular” region, also known as the Virchow-Robin space, is defined as the surrounding space of penetrating arterioles constrained by the basement membranes of astrocytic end feet and the basement membranes of arterial smooth muscle cells, mediating a fluid drainage system (Bacyinski et al., 2017; Diem et al., 2018; MacGregor Sharp et al., 2019). The convective influx of CSF into the brain coupled with the activity of Aquaporin-4 (AQP4) expressed on astrocytic end feet appears to drive ISF toward these paravascular spaces.

Drainage of ISF may lead to an osmotic pressure gradient that allows for an AQP4 mediated CSF exchange mechanism (Pirici et al., 2018). This recycling of fluid facilitated by astrocytic glial cells in paravascular regions of the brain defines the “glymphatic” system (Iliff et al., 2012). It is held that the glymphatic circulatory pathway, serves to clear extracellular solutes, proteins, and metabolites as a primary drainage mechanism against potentially pathological accumulations (Iliff et al., 2012). In spite of this, glymphatic circulation alone fails to explain the deposition of amyloid-beta (Aβ) peptides in the walls of arteries as cerebral amyloid angiopathy, a key feature of aging and Alzheimer’s disease (AD; Keable et al., 2016). Experimental work and observations on post-mortem human brains determined that ISF also drains out of the brain along the basement membranes of capillaries and basement membranes surrounding smooth muscle cells as intramural periarterial drainage (IPAD; Albargothy et al., 2018). This “perivascular” compartment, an area bound by the middle layers of the basement membrane within the arterial tunica media, is a contributor to fluid homeostasis in the brain (Bacyinski et al., 2017). As a result, revealing the complexity of the CSF and ISF circulation and clearance is of great relevance to understanding the physiological homeostasis of the brain (Carare et al., 2020). This review will focus mainly on CSF.

## Animal Studies

**Figure 1:**
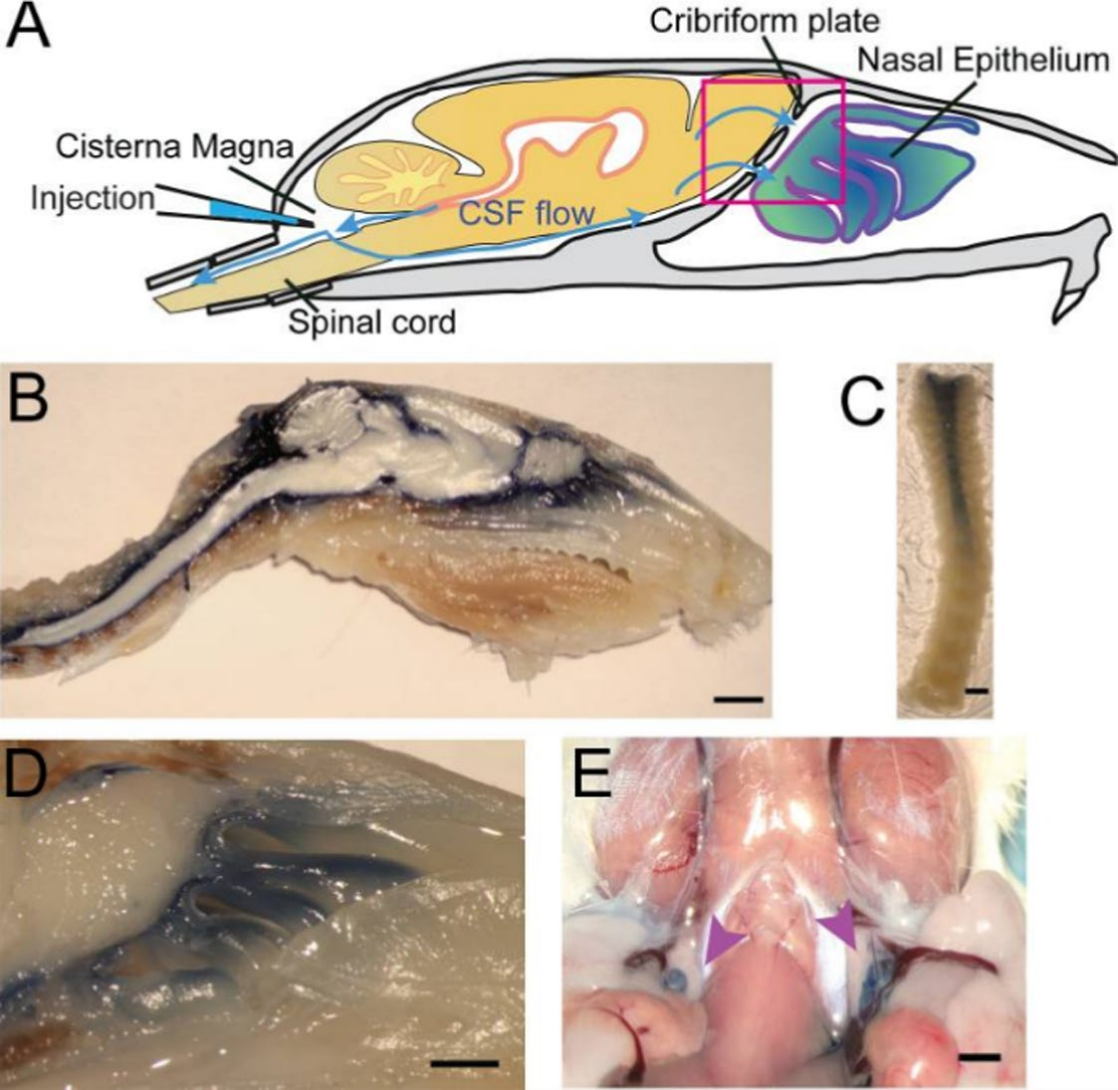
Structural depiction of CSF flow through the cribriform plate of a mouse model from Norwood Et. Al. (PMID: 31063132). **(A)** Schematic diagram of anticipated egress from neural tissue into nasal epithelium. **(B)** Sagittal midline section following injection of EB into cisterna magna. **(C)** Decalcified spinal column after EB injection into cisterna magna. **(D)** Visualization of the area enclosed by pink box in **(A)** depicting contrast movement across the cribriform plate. **(E)** EB dye localization into deep cervical lymph nodes marked by purple arrows following injection at cisterna magna.

Our understanding of CSF egress pathways is evolving. This review will focus on the brain-nose-environment interfaces whose data stem largely, but not exclusively, from tracer studies in a variety of animal models. In mice, CSF drainage systems can be visualized through injection of high-contrast Evan’s blue (EB) into the cisterna magna. In the CSF, EB remains unbound to proteins and can act as a low-weight molecular tracer transported by fluid convectional pathways. Permeation of EB into the nasal lymphatic system provides evidence for drainage of CSF through the cribriform plate (CP) in mice (Norwood et al., 2019). Enriched EB detection in the nasal epithelium implicated the structural importance of the cribriform plate (CP) in CSF fluid dynamics and added to a growing repertoire of animal studies that collectively pointed to a critical connection between fluid flow around the neural tissues of the olfactory bulb and nasal lymphatic drainage. Further examination in the rat model using India ink also established a direct link between structural channels through the cribriform plate into the nasal submucosa (Kida et al., 1993). CSF tracing studies focused on the nasal cavity drainage in the rodent model linked the lymphatic vessels embedded in the nasal mucosa with the subarachnoid space *via* the CP (Nagra et al., 2006; Aspelund et al., 2015; Hsu et al., 2019). However, evidence for the involvement of a cribriform plate-mediated nose-brain connection in CSF flow is not limited to the murine model (Figure 1).

A similarly proposed mechanism of CSF outflow across the cribriform plate has been extensively studied in adult and neonatal sheep. Injection of ^131^I-Human Serum Albumin (HSA) into the cisterna magna resulted in an increased radioactivity within the nasal mucosa, nasal conchae, and nasal septum, reinforcing the view of a cribriform mediated nose-brain connection, predominant in fetal and neonatal life (Papaiconomou et al., 2002). Further experiments that artificially obstructed CSF movement through the cribriform plate demonstrated large increases in intracranial fluid pressure, thus validating in mammals the nasal drainage pathway as fundamental in the physiological clearance of CSF (Silver et al., 1999; Mollanji et al., 2002; Papaiconomou et al., 2002). Examinations of ventricular fluid turnover in rabbits by tracing the movement of CSF also reaffirms a nose-brain model that implicates the cribriform plate boundary (McComb et al., 1982; Bradbury and Westrop, 1983).

In a series of tracer-based cadaveric studies, Johnston et al. investigated nasal CSF outflow in monkeys, mice, rats, pigs, and humans using yellow MicroFil injections into the subarachnoid space. Following infusion of the tracer, overall, the findings displayed a circulation of MicroFil into the nasal region across species, and tracer was detected in both the nasal mucosa and nasal turbinates (Johnston et al., 2004). While the characterization of CSF drainage through the nasal mucosa is of crucial consequence, the extent to which post-mortem observations can extrapolate to normal physiological conditions remains unclear (Johnston et al., 2004). Ultimately, the synthesis of information gathered from a variety of animals led to the acceptance of the nose-brain connection in Cerebrospinal Fluid efflux mechanisms. Despite this, the increased complexity and physiological nuance afforded by human anatomy may limit the application of our animal-based understanding of CSF circulation to humans. In this manner, while the characterization of CSF circulation in animals provides a concrete groundwork, direct analysis of CSF in humans (post-mortem and *in-vivo*) must be used for the proposed existence of a similar connection in humans.

## Importance of the Nasal Pathway Across Species

Perhaps, the most significant uncertainty of the olfactory drainage route for CSF in humans revolves not around its anatomical existence, but rather the extent to which this pathway is utilized. In a variety of animal models, passage through the cribriform plate and into the lymphatic system of the nasal mucosa defines a major pathway for CSF efflux. In sheep, for example, mathematical analysis of intracranial pressure after blocking the cribriform plate allows for an estimation of the fraction of CSF that proceeds through a nasal elimination route (Boulton et al., 1997). Similar quantifications in animal models allow for a straightforward investigation of the fractional importance of an olfactory drainage route when compared to other established clearance pathways (Table 1).

**Table 1:** Comparative analysis of olfactory pathway.

Species	Study	Approximate % of CSF drained through olfactory route	Methodology of CSF Tracing	Duration of Observation
Sheep	Boulton et al., 1997	>50%	Intraventricular injection of Human Serum Albumin	6 h
	Silver et al., 2002	>50%	Intercranial Pressure calculation following CP blockage	300 s
Rats	Boulton et al., 1999	40–50%	Intraventricular injection of Human Serum Albumin	6 h
Rabbits	Bradbury et al., 1981	40–50%	Intraventricular injection of radioiodinated albumin	6 h
Dogs	Sokołowski et al., 2018	10%	Volumetric analysis of the rostral cranial fossa	N/A

*Synthesized comparison of proportional CSF egress occurring by means of an olfactory drainage route as quantified by* Bradbury et al., 1981, Boulton et al., 1997, 1999, Silver et al., 2002 *and* Sokołowski et al., 2018.

The cribriform plate-mediated drainage pathway found in many animals is a major but variable route of CSF drainage. An exact assessment of the proportional flow of CSF through nasal lymphatics in humans, however, remains unknown. A significantly less pronounced fraction of CSF cleared *via* an olfactory path in humans may provide an explanation for the difficulty experienced in visualization and quantification of CSF traversing the cribriform plate into nasal lymphatics (see below). Emerging as an extension of work based on various animal models, the significance of a human nasal clearance route remains fundamentally unsolved.

## Csf Clearance Mechanisms

The balance between CSF production and clearance regulates intracranial pressure (ICP), and abnormalities of this system are believed to underlie conditions, such as normal pressure hydrocephalus (See “Human Pathology”). Classically, the elimination of CSF in the human was believed to be *via* one-way valves (Pacchionian (arachnoid) granulations) penetrating the dura and delivering CSF and ISF to circulating venous blood. This absorption along arachnoid granulations was believed to be along the superior sagittal sinus (SSS; Davson et al., 1973; Brinker et al., 2014; Khasawneh et al., 2018) and spinal nerve root sleeves, the latter draining into spinal radicular veins (Tubbs et al., 2007; Ma et al., 2019). More recently, increased imaging capacity and extensive animal study have yielded a growing understanding of the circulation of CSF into the lymphatic system, a network of vessels that aide in CSF and waste removal.

**Figure 2:**
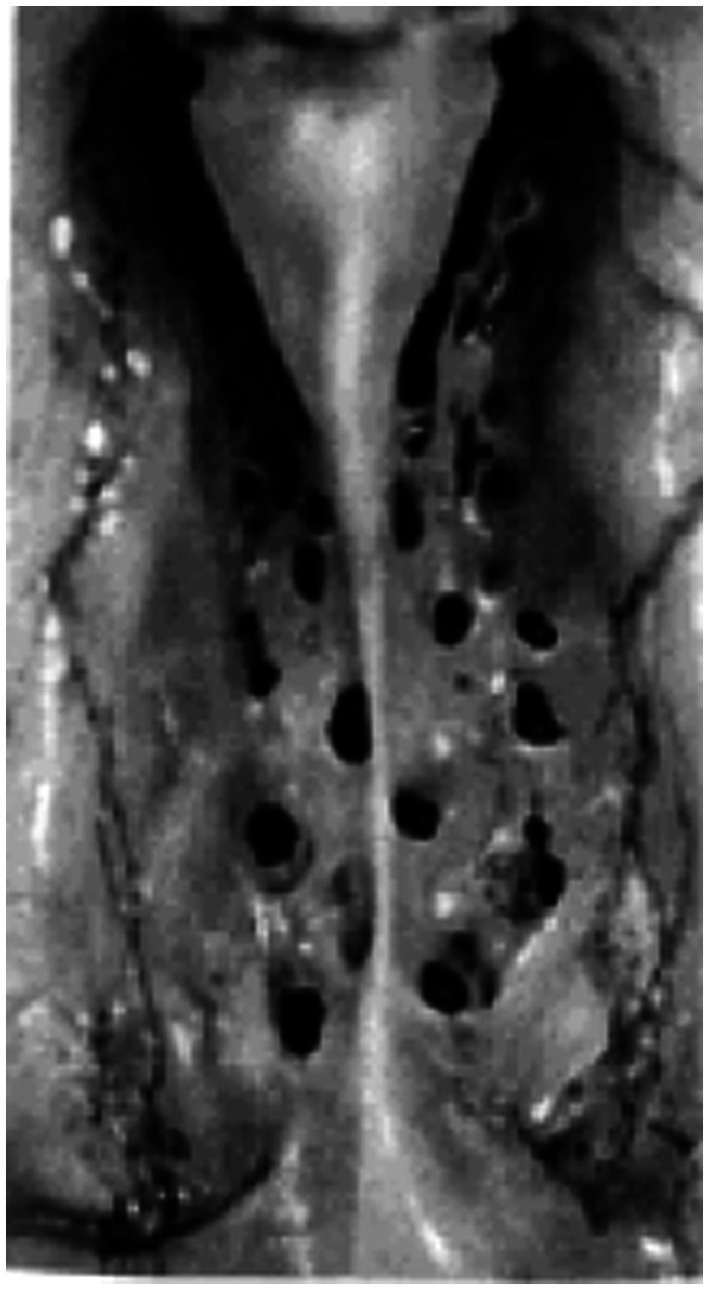
Photographic image of human cribriform plate. Superior view of the cribriform plate (left and right halves) in a 25-year-old female subject from Kalmey et al., (1998).

Aside from the classical conception of CSF clearance along arachnoid granulations, the nuances of CSF drainage into the lymphatic system remain highly contended (Proulx, 2021). Recent evidence highlighting the existence of lymphatic vessels in the dura mater of the mouse brain supports a CSF drainage mechanism leading to deep cervical lymph nodes *via* dural lymphatics (Aspelund et al., 2015). Discussion of a potential efflux of CSF through dural lymphatic vessels in the skull and spine has been contextualized to a growing literature that also examines the role of the cranial nerves as a drainage site into cervical lymphatics (Sakka et al., 2011; Brinker et al., 2014; Chen et al., 2015; Ma et al., 2019). Among these cranial nerves, the clearance of CSF along the olfactory nerve into nasal lymphatics based widely on animal studies remains inadequately studied in humans (Figure 2).

## Post-Mortem Human Studies

**Figure 3:**
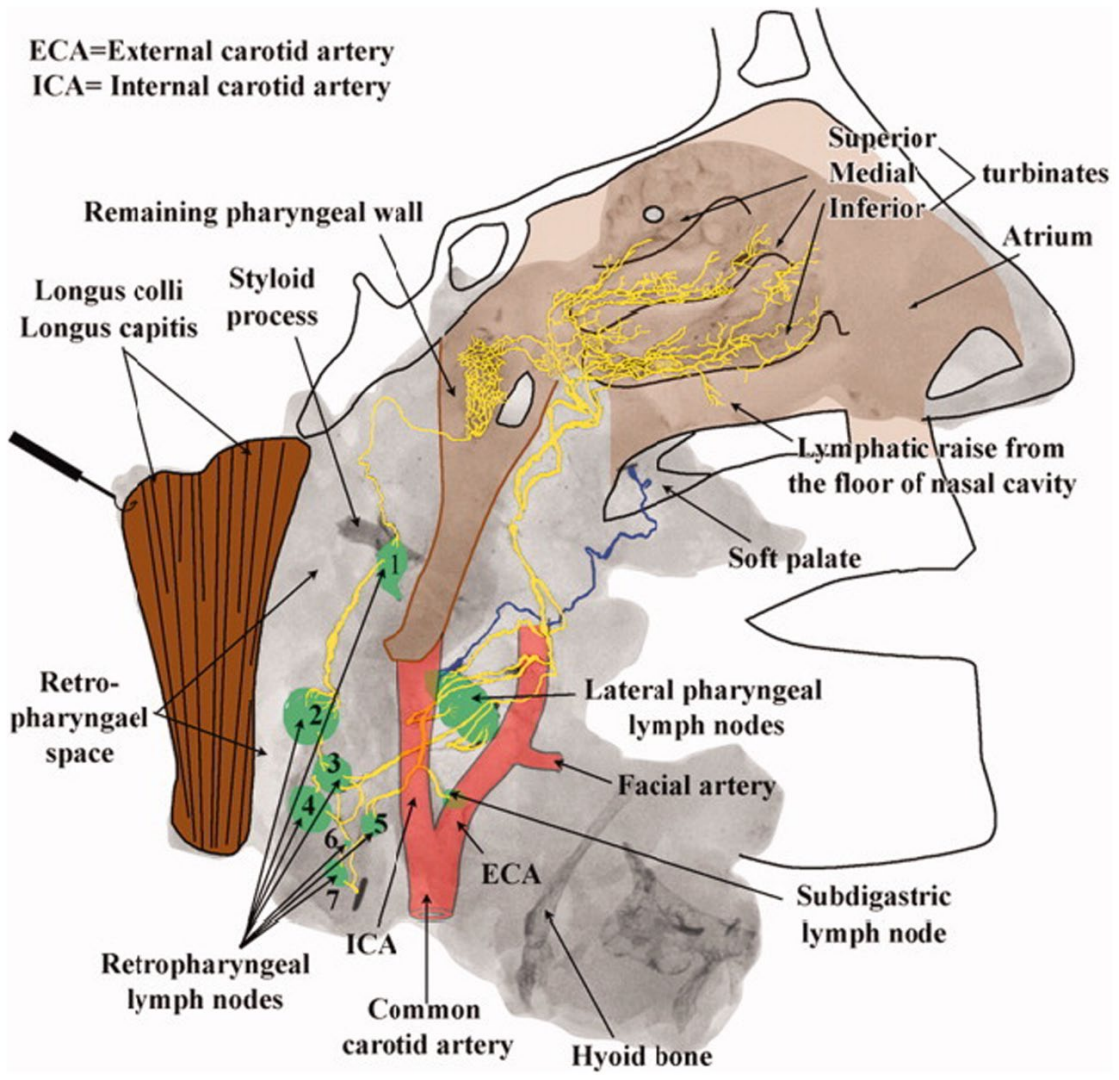
Lymphatic drainage of the nasopharyngeal region. Radiographic image from Pan et al. mapping lymphatic drainage from point of tracer injection near nasal turbinates to the cervical lymph nodes of the upper neck. Presumed lymphatic vessels drain into the lateral pharyngeal (level II) and retropharyngeal lymph nodes demonstrating an extensive lymphatic network extending from the nasal mucosa to the upper neck (Pan et al., 2009).

The existence of an olfactory clearance mechanism across multiple animal species suggests a similar physiology in humans. In one post-mortem radiographic study of nasal lymphatics, four halves of skulls and necks obtained from fresh human cadavers were injected with a suspension of radio-opaque lead oxide and powdered milk at an inflated initial lymphatic vessel of the inferior turbinate using a surgical microscope (Pan et al., 2009). Following injection, specimens were photographed, radiographed, and analyzed for lymphatic pathways in the nasal region. The results displayed an extensive capillary network of lymphatic drainage beginning along the superior, medial, and inferior nasal turbinates that flows largely into the lateral pharyngeal and retropharyngeal lymph nodes (Pan et al., 2009). The radiographic evidence of “a rich avalvular and well-organized lymph capillary network” in the nasal mucosa seems to anatomically confirm the possibility of a nose-brain connection for CSF efflux in humans (Pan et al., 2009; Figure 3).

In another cadaveric study, immunohistochemical exploration of the deep cervical lymph nodes was conducted in patients who died from intracerebral hemorrhage (Caversaccio et al., 1996). Results indicated a statistically significant increase in iron prevalence within lymph nodes associated with intracerebral hemorrhage, as estimated by Prussian blue staining. These findings of lymphatic drainage through the nasal region strongly suggest a physiological connection between the brain and the lymphatic system of the upper cervical region (Caversaccio et al., 1996). An additional post-mortem analysis of CSF efflux pathways and cellular clearance examined eighteen autopsies with varied pathologies alongside 6 control autopsies from neurologically normal patients (Lowhagen et al., 1994). To examine the location of drainage of red corpuscles and iron in patients with intracranial hemorrhagic lesions, samples were stained with Prussian blue (Lowhagen et al., 1994). Results indicated an aggregation of red corpuscles in the nasal mucosa in cases of recent subarachnoid hemorrhage, and in older lesions displayed iron-containing pigment in the nasal mucosa. For hemorrhagic lesions, the findings suggested a clearance pathway from the subarachnoid space into the nasal mucosa through a perineural pathway (Lowhagen et al., 1994). In this project, three additional specimens were also examined using an intracranial injection of India ink into the olfactory groove. Visualization of India ink penetration reveals an explicit egress pathway for CSF into the nasal mucosa (Lowhagen et al., 1994). Post-mortem analysis of fluid dynamics in the olfactory region using India ink by Lowhagen et al. thus seems to support the presence of a nasal pathway for CSF drainage in humans. Much more recently, mucosal analysis of α-synuclein aggregates in the olfactory region revealed increased diagnostic sensitivity in Parkinson’s disease and REM sleep behavior disorder patients (RBD; Stefani et al., 2021). RT-QuIC, real-time quaking-induced conversion, detection of α-synuclein aggregates displayed a mucosal positivity of 44.4% for RBD patients, 46.3% for Parkinson’s patients, and 10.2% for control subjects (Stefani et al., 2021). Ultimately, the detection of α-synuclein aggregation is comparable to corpuscle aggregation in olfactory mucosa and deepens our understanding of the physiological and pathological relevance of fluid drainage across the brain-nose interface (Hsu et al., 2021a).

**Figure 4:**
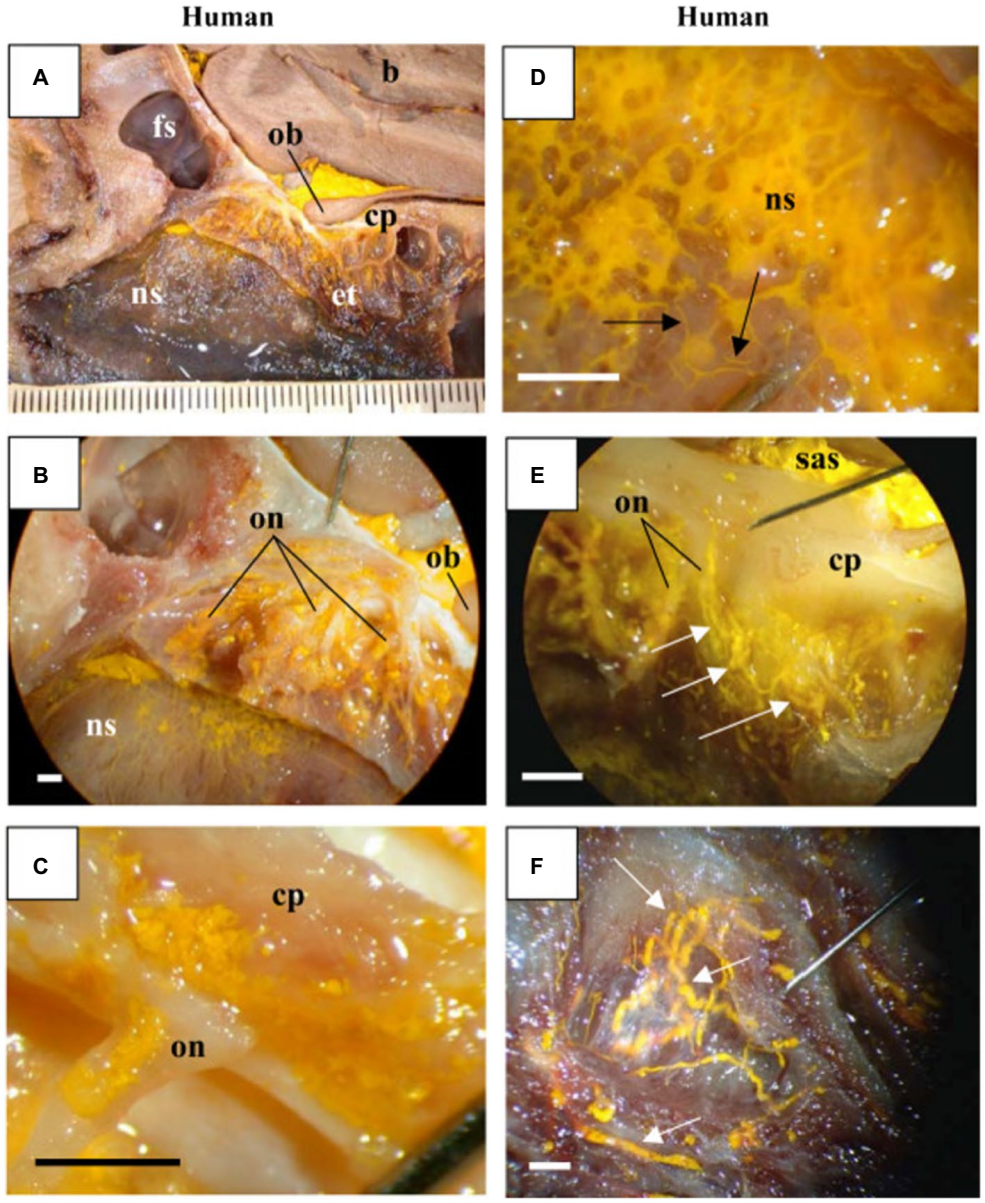
MicroFil penetration across the cribriform plate: Visualized results using sagittal cross sections of human cadaveric sample from Johnston et al., (2004) displaying yellow MicroFil penetration into the nasal region from subarachnoid space. **(A)** Drainage around olfactory bulb across the cribriform plate into the ethmoid turbinates and nasal septum. **(B,C)** MicroFil detection around the perineural spaces of the olfactory nerve. **(D)** Close up visualization of lymphatic drainage in the nasal septum. **(E)** MicroFil permeation from the subarachnoid space into the ethmoid labyrinth. **(F)** Enhanced display of lymphatic drainage in superior nasal turbinate. Key: Brain (b), frontal sinus (fs), olfactory bulb (ob), cribriform plate (cp), ethmoid turbinates (et), nasal septum (ns), olfactory nerve (on), and subarachnoid space (sas).

Characterization of CSF drainage through the nasal mucosa was first examined in Johnston et al., whose experimental design utilized MicroFil injections into the subarachnoid space across a variety of species (described above). These studies also included one human sample (Johnston et al., 2004). Here, for the one human cadaver examined, MicroFil was found in both the nasal mucosa and nasal turbinates (Johnston et al., 2004). This post-mortem finding provided the first direct visualization of a CSF clearance pathway across the cribriform plate and into the olfactory region, implicating the brain-nose interface in CSF dynamics. While the characterization of CSF drainage through the nasal mucosa is of crucial consequence, the extent to which post-mortem observations can extrapolate to normal physiological conditions is unclear (Johnston et al., 2004). The penetration of MicroFil directly into the lymphatic draining vessels was obscured in the human, as the tracer was diffusely found in the nasal interstitium. It is not entirely clear; however, whether tracer applied in the subarachnoid space of a cadaver is capable of replicating the living physiological conditions of CSF transport in the nasal region that may be interconnected with blood flow. Contrary to the animal subjects however, this lack of lymphatic permeability to MicroFil in humans could also likely be explained by a degradation of tissue that occurs rapidly following death (Johnston et al., 2004; Figure 4).

Overall, the results from multiple post-mortem studies provide evidence for the clearance of Human CSF across the cribriform plate and into the nasal mucosa and cervical lymphatic circulation. Despite this, post-mortem work fails to adequately model the pressure dynamics and cellular mechanics of fluid flow that contribute to CSF outflow pathways of living humans. The importance of steady-state pressure equilibria on fluid dynamics, active cellular and vascular processes on CSF movement, and undeniable changes to histology following death are unaccounted for in these post-mortem examinations. On an experimental level, the technique of pushing contrast agents under abnormal pressure conditions in post-mortem samples leaves open the question of whether these studies can accurately assess CSF movement and pathways in living humans. Ultimately, while these cadaveric observations may suggest the possibility of CSF egress through the nasal region into the lymphatic system, they must be confirmed by *in-vivo* imaging studies that physiologically demonstrate the importance of the anatomical pathway.

## *In-vivo* Human Studies

One examination of human subjects using lymphoscintigraphy coupled with radioactive gold injection into the nasal turbinates revealed a vascular system of lymphatic drainage that extended from the nasal turbinates into cervical lymph nodes (Fernández et al., 2005). This study, based on the unilateral injection of radioactive tracer into the head of the inferior nasal turbinate in 30 patients diagnosed with Otitis Media (inflammation of the middle ear), provided evidence of a lymphatic network clearing human CSF across the nasal epithelium and into deep cervical circulation (Fernández et al., 2005). An *in-vivo* examination utilized MRI with time-of-flight angiography sequences to elucidate the directionality of flow within meningeal lymphatic vessels (MLV) running alongside the SSS. A coronal section anterior to the vertex of the skull was used to image meningeal lymphatic vessels running parallel the SSS. The results for the six participants examined indicated CSF flow in MLV was countercurrent to venous flow along the SSS (Kuo et al., 2018). The counter directionality of lymphatic flow shows a CNS lymphatic drainage *via* the cribriform plate and supports the hypothesis that the olfactory region is involved in fluid egress from the CNS (Kuo et al., 2018).

Further evidence for nasal clearance of CSF comes from a study that examined iron concentrations in nasal exudate following acute stroke (Garcia-Cabo et al., 2020). A non-invasive nasal swab was used within the first 48 h following a cerebrovascular accident, and iron content was measured using inductively coupled plasma-tandem mass spectrometry (Garcia-Cabo et al., 2020). Significant differences in variability of iron concentration between hemorrhagic and ischemic patients measured using the interquartile range found a 15-fold increase in the hemorrhagic group (Garcia-Cabo et al., 2020). Analysis of nasal exudate samples illustrated a pathway from the CNS to the nasal cavity that can serve as a clearance site for pathologically relevant biomarkers, in this case Iron, that helped differentiate between ischemic and hemorrhagic stroke (Garcia-Cabo et al., 2020). While this finding reaffirms the significance of the nose-brain interface, it fails to directly confirm the role of nasal lymphatics in normal CSF clearance.

While the exploration of lymphatic drainage (Fernández et al., 2005), countercurrent fluid dynamics (Kuo et al., 2018), and nasal biomarker clearance (Garcia-Cabo et al., 2020) all contribute to a growing awareness of the potential for CSF clearance along an olfactory route, they fail to specifically characterize the drainage of CSF across the cribriform plate in real time. Accordingly, *in-vivo* confirmation of the nasal route for CSF outflow must also incorporate imaging studies (MRI/PET) that utilize a variety of tracers that can monitor the flow of CSF as it circulates, and precisely address the pathways by which it clears.

Extended to humans, MRI tracing of CSF bulk flow using intrathecally administered gadobutrol, a low-molecular weight contrast agent that can help monitor CSF flow in real time, displays direct involvement of the parasagittal dura, further suggesting a connection between dural lymphatics and CSF flow along the subarachnoid space (Ringstad and Eide, 2020). Recent advances in neuroimaging coupled with the discovery of dural lymphatics have revived interest in better understanding the exact mechanisms by which CSF efflux and lymphatic drainage may be related (Proulx, 2021). Accordingly, a push to characterize and quantify the clearance of the CSF through a nasal pathway has bolstered a growing repertoire of post-mortem and *in-vivo* imaging studies that attempt to clarify the flow of CSF across the cribriform plate in humans.

**Figure 5:**
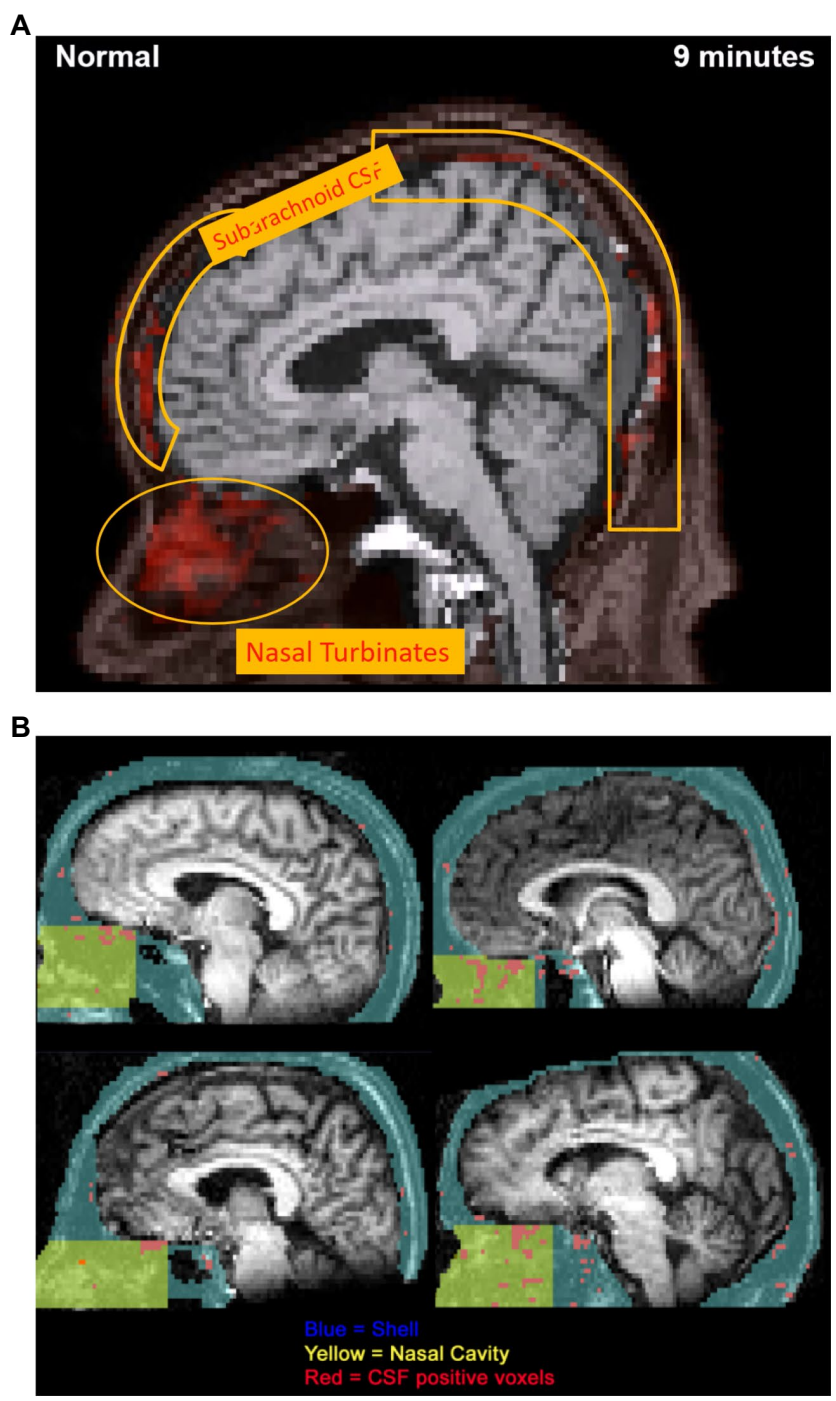
**(A)** ^18^F-THK5117 PET image obtained from supplementary video visualizing extracranial CSF transport over 9 min. Progressive 1 min temporal offsets between time-activity curves for the ventricles and individual voxels were used to highlight the anatomical movement of CSF over 9 min. PET image at 9 min displays the clearance pathway of CSF-positive voxels from the subarachnoid space to the nasal turbinates, demonstrating an olfactory clearance mechanism. This research was originally published in *JNM* (PMID: 28302766). **(B)** ^11^C-cocaine PET data obtained using 40 min duration of a CTI-931 tomograph following injection of 6–8 mCi of ^11^C-cocaine. Images from normal control participants depict CSF-correlated voxels (r > 0.95) mapped in red. Nasal cavity region (yellow) is defined within a larger shell (blue), and CSF-positive voxels were traced into the olfactory region. Superior and middle turbinate regions provided the highest density of CSF-correlated voxels (materials derived from Dr. Nora Volkow NIDA and reported in de Leon et al., 2017 PMID: 28302766).

**Figure 6:**
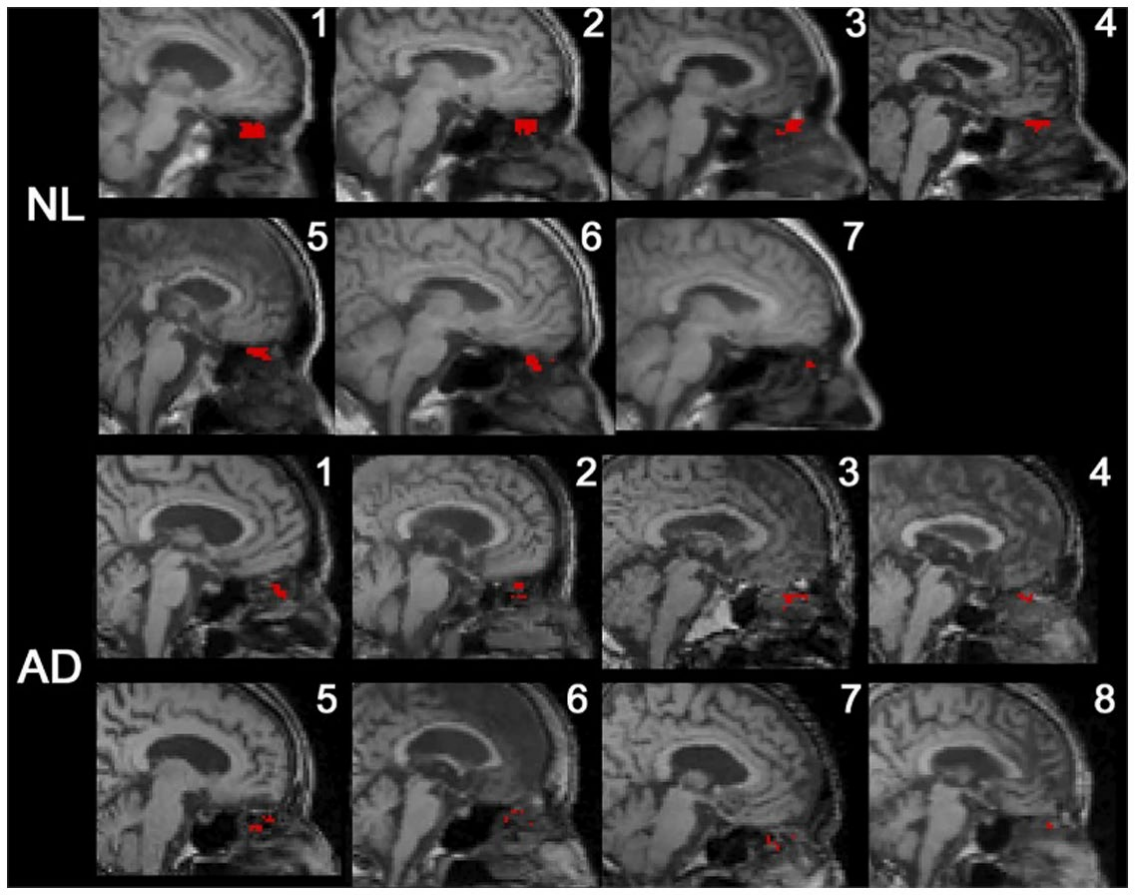
^18^F-THK5117 PET data superimposed on T1-weighted MR images depicting CSF-positive voxels in the superior nasal turbinate mapped in red. CSF signal was depicted in all AD subjects and control. The data showed 65% fewer CSF-positive voxels in the superior turbinate of AD subjects (de Leon et al., 2017).

We recently investigated CSF efflux pathways in Alzheimer’s disease and control subjects using dynamic PET imaging of ^18^F-THK5117, an intravenously administered tau pathology tracer (de Leon et al., 2017). Eight subjects diagnosed with AD and seven control participants were examined with PET and T1-weighted MRI. MR imaging and corresponding PET scans were coregistered, and the time-activity curve of the ventricular CSF was used to identify positive voxels throughout the brain. The data showed CSF-positive voxels in the nasal cavity and the subarachnoid and basal cisterns (see Figure 5A). In this paper’s supplemental data, it was also reported, using ^11^C-cocaine tracer-based PET imaging, that young adult subjects also showed a nasal CSF clearance pathway (see Figure 5B). The ^18^F-THK5117 PET data demonstrated that reduced CSF clearance at both the lateral ventricle and superior nasal turbinates separated the AD from the control group (see Figure 6). Moreover, this study also showed that CSF clearance was highly associated with brain amyloid β (Aβ) deposits as determined by ^11^C-PiB-PET scan (de Leon et al., 2017). These PET imaging data provide additional *in-vivo* anatomical and disease-related evidence for a physiological CSF pathway exiting from the cribriform plate, through the nasal turbinates, and into the superior turbinates. These results also offer support for an impaired CSF clearance relationship contributing to the accumulation of brain amyloid.

The demonstration of the nasal pathway for CSF clearance of humans using intravenous administration of^18^F-THK5117 and ^11^C-cocaine-based PET imaging is complemented by the study of the olfactory CSF connection using intrathecally administered gadobutrol-based imaging. An *in-vivo* MRI study examined the time course of CSF using intrathecally administered gadobutrol contrast material in 19 patients with various CNS disorders (Eide et al., 2018). Imaging of the head and neck took place at baseline at up to five time points, approximately 3,5,8,24, and 48 h post-contrast. The peak CSF signal enhancement was 5–8 h after injection. The peak activity in lymph nodes took place at 24 h, which coincided with the peak gray matter signal. The latter was interpreted as glymphatic signal enhancement (Eide et al., 2018). The authors interpreted the lack of direct nasal enhancement on MR images coupled with a late peak in neck lymph nodes as not indicative of CSF drainage through nasal lymphatics *via* olfactory perineural routes in the human (Eide et al., 2018). This group demonstrated with the same MR methods, in an additional study, the role of perineural CSF drainage in 24 cases of varying CSF disorders (Melin et al., 2020). Contrast enhancement was again not found in the nasal mucosa of the superior, middle, or inferior turbinates (Melin et al., 2020). Despite this negative finding, significant tracer enhancement was found inferior to the cribriform plate in 11/24 patients, which may represent egress along perineural spaces across the cribriform plate (Melin et al., 2020).

As such, the existence and significance of CSF clearance *via* the nose-brain interface remain uncertain. In the MRI studies, the intrathecally administered gadobutrol imaging questions the universality of the brain-nose pathway that are suggested by the post-mortem and PET imaging work. This disparity could be methodological, perhaps explained by the increased tracer contrast sensitivity afforded by PET or by the different scan times examined after the tracers were administered. Nevertheless, these observations raise questions regarding the magnitude and potential importance of this pathway in the human. The anatomic potential for nasal clearance of CSF may simultaneously be counterbalanced by a sharply reduced utilization of this pathway in humans. It remains to be proven whether there are species differences between humans and the many murine and mammal animal models studied.

## Human Pathology

It has been hypothesized that reduced CSF egress along an olfactory route is related to the pathogenesis of Alzheimer’s disease, by promoting the intraparenchymal accumulation of Aβ, a key pathological feature of AD (Ethell, 2014). In mice of varying age, fluorescent imaging of intracisternally injected albumin revealed a negative correlation between CSF clearance rate and age, providing a potential explanation for the age-related prevalence of neurodegenerative disease (Brady et al., 2020). An important consideration in the pathogenesis of sporadic AD appears to be an imbalance in the production and clearance of Aβ that is linked to IPAD, glymphatic, and CSF clearance mechanisms in humans (Hardy and Selkoe, 2002; Tarasoff-Conway et al., 2015). The pathological significance of reduced CSF clearance was examined in a quantitative study of CSF production rates in patients diagnosed with various neurodegenerative diseases. The data reported showed a significantly decreased CSF flow in AD patients (Silverberg et al., 2001).

While the measurement of human CSF dynamics is still rudimentary, an understanding of CSF clearance mechanisms is increasingly important to a variety of diseases. For years, this has been a consistent topic of investigation in normal pressure hydrocephalus (NPH; Bradley, 2015). NPH is a form of communicating hydrocephalus where alterations in CSF flow and clearance, rather than physical obstruction, lead to transient pressure waves that cause neuronal dysfunctional and typical symptoms of gait instability and memory disorder. *In-vivo* examination of CSF movement using Gadobutrol revealed delayed clearance in patients with NPH, suggesting a relationship between drainage mechanisms and NPH pathogenesis (Eide and Ringstad, 2019). Furthermore, CSF shunting may even help counter the intracerebral accumulation of Aβ associated with NPH (Moriya et al., 2015). Given the lack of clear understanding of the causes of NPH, as well as an absence of objective markers to reliably identify patients who may respond to surgical intervention, understanding CSF fluid dynamics and clearance mechanisms, including novel mechanisms, such as nasal clearance, provide opportunities for better diagnosis and treatment of NPH. More recently, a growing understanding of SARS-Cov-2 progression and diagnosis reveals a potential connection between COVID-19 biomarkers and CSF clearance (Edén et al., 2021; Lewis et al., 2021). Overall, clarifying the existence of an olfactory route for CSF clearance with peripheral CSF detection in humans may yield a practical and cost-effective clinical means of detecting pathologies whose biomarkers can only be measured in CSF (Liu et al., 2018).

## Nose-Brain Interface: Immunology

The nasal turbinates of the upper respiratory tract serve as a common point of entry for many viruses, bacteria, and toxins to the brain (van Riel et al., 2015). Accordingly, within the nasal cavity, olfactory sensory neurons (OSN) in direct contact with the outside environment are exposed to pollutants and infectious agents in the air. Lining the olfactory epithelium, the hundreds of thousands of olfactory sensory neurons offer these viruses access to the brain *via* an olfactory nerve route through the cribriform plate and olfactory bulb (Moseman et al., 2020). Consequently, exposures to viruses including Sindbis, herpesvirus, pseudorabies, influenza, polio, Borna, rabies, and environmental toxins including lead, manganese, solvents, and pesticides may lead to their entrance to the brain where they can induce inflammation, necrosis, and degradation of neuronal tissue (Van den Pol et al., 2014). Recent studies demonstrate the route of entry into the brain of SARS-CoV-2 virus along olfactory neurons by axonal flow and through tight spaces between ciliated cells in the olfactory epithelium (Ylikoski et al., 2020). The accessibility of the environment-nose-brain connection to a potentially pathologic microbiome can also be extended to the clinical diagnosis for neurodegenerative disease. In the case of Parkinson’s disease, Lewy-type α-synucleinopathy occurring in the olfactory bulb is strongly indicative of PD prognosis, implicating the environment-nose-brain interface in a clinical context (The Arizona Parkinson’s Disease Consortium et al., 2009). While understanding the specific mechanisms associated with pathogen entry and nasal drug delivery are beyond the scope of this paper, the clinical relevance of the environment-nose-brain pathway is of importance to both the diagnosis of neurodegenerative disease *via* CSF biomarkers and the delivery therapeutic products/drugs that can otherwise not readily bypass the blood–brain barrier. In this manner, the immunological and CSF clearance mechanisms of the olfactory region potentially reveal essential functions that warrant continued research into the understanding of the environment-nose-brain interactions in human diseases.

With a rich microbiome populating the nasal cavity, resident bacteria can also gain access to the brain (Zhou et al., 2014; Kumpitsch et al., 2019) as can proinflammatory cytokines (Hsu et al., 2021b). Imbalances in bacteria, referred to as dysbiosis and the associated host immune responses, have been implicated in upper respiratory chronic inflammatory diseases (Wagner Mackenzie et al., 2017; Lee et al., 2018). The inflammatory/dysbiotic environment may facilitate bacterial access to the subarachnoid space through the cribriform plate. Periodontal research also suggests that oral bacteria or inflammatory biproducts can access the brain *via* both blood and neuronal routes (Riviere et al., 2002) and our studies suggest oral bacteria can induce amyloid pathology (Riviere et al., 2002; Kamer et al., 2015, 2021). As such, bacterial DNA whose sources could be from the gut and respiratory pathways or proinflammatory cytokines may access the brain *via* an olfactory route at the nose-brain interface. Experimental evidence for this route comes from the study of *C. pneumoniae*, an intracellular bacterium colonizing the mucosa of the upper respiratory tract. In an animal model, following nasal infection with *C. pneumoniae*, antigens were identified in the olfactory neuroepithelia and olfactory bulbs (Balin et al., 2018). This finding suggests a direct communication between the nasal region and CNS that may allow for nasal/oral bacterial infection. Bacteria-induced permeability of olfactory epithelium could potentially enhance this communication. The potential for bacterial infection of the CNS *via* an olfactory connection may also contribute to amyloid pathology through decreased CSF clearance across the cribriform plate (Kamer et al., 2000).

The immune function of the nose-brain connection plays a crucial role in blocking and targeting harmful pathogenic toxins that threaten host health. Firstly, several neuroimmune processes are triggered by invading viruses which are characterized by an innate, proinflammatory immune response (Tonelli and Postolache, 2010). The human nasal mucosa, a mucous membrane lining that spans from the nasal turbinates to the pharynx, activates a series of defense mechanisms against viral invasions supported by antigen-presenting cells and lymphocytes (Wright et al., 2012). Secondly, the stimulation of an antigen-specific “peripheral” immune response is essential in achieving complete (or near) viral clearance. In a study centered around the cellular immune defense mechanisms toward *Streptococcus pneumoniae*, an upper respiratory colonizing bacterium, samples of nasal wash were taken from infected individuals and measured for an altered presence of immunoglobulin-specific antibodies (IgG and IgA). Using an ELISA assay and Western blotting analysis, anti-protein expression of IgG and IgA against *S. Pneumoniae* was found to be boosted in nasal wash after intranasal application of *S. Pneumoniae*, suggesting that the adaptive immune system is of importance to the environment-nose-brain interface (Wright et al., 2012). Specific immune responses also include the assembly of T-lymphocytes and creation of type I interferons, and cytokines responsible for viral destruction and limiting infection spread (Tonelli and Postolache, 2010).

## Discussion

The evidence for an olfactory route of clearance in humans began as an extension of the prevalent understanding of CSF fluid dynamics in animal models, progressed into post-mortem evaluations and now revolves around various *in-vivo* imaging modalities. While there exists a growing repertoire of imaging techniques that place an increased importance on nasal involvement in CSF egress, the existence and significance of this pathway remain unsettled in a modest, complex, and contradictory literature. The inconsistency of tracer enhancement recorded by PET and MRI reveals the need for an expanded examination of the nose-brain connection using multiple imaging techniques. Recent developments in the use of multi-modal imaging modalities, including PET/MR, may provide the technological capacity to reveal the basis for the inconsistent observations of CSF clearance through the nose in humans (Bailey et al., 2015). Understanding the impact of tracer molecular weights and tissue affinities on the efficacy of MRI and PET tracers and expanding the range of contrast agents used to image CSF flow, will help improve the understanding of olfactory CSF egress. While evidence examining the physiological nuances of CSF efflux through the nose continues to expand, further insight into the volumetric magnitude of fluid flow through such a pathway must also be adequately quantified. Given the recency and novelty of *in-vivo* CSF mapping, the pathological considerations of CSF egress and the extent of nasal involvement in the proposed pathway require further study. Study of the olfactory drainage route for CSF in humans may permit measurement of brain specific biomarkers in nasal exudates, including neuronal proteins, such as tau protein, and may increase the sensitivity for identification of prostaglandin D2 synthase (β-trace protein), an established CSF-leak marker (PMID: 27614217). Although the animal models provide a reliable, accessible, and ethically sound means of assessing the structural pathways of CSF movement, they fail to encapsulate the physiology of human CSF outflow. *In-vivo* human studies become paramount. Understanding the role of the nose-brain connection in clearance of CSF from the nervous system may provide diagnostic assistance in infectious disease, NPH, CSF leaks, and age-related neurodegenerative diseases. Ultimately, the broadening scope of physiological research will continue to allow us to better understand the importance of the nose-brain connection to CSF clearance mechanisms in humans.

## Author Contributions

NHM and MDL conceptualized the review. NHM is the principle author of the manuscript. All authors are contributing authors. MDL is the corresponding author for this review.

## Funding

This review was partially supported by the following research grants: R01 AG12101, R01 AG022374, R01 AG13616, RF1 AG057570, R56 AG058913-MdeL., R01AG068398-GCC., R56 NS111052-TB, and-R01 AG057848-YL. KB is supported by the Swedish Research Council (#2017-00915), the Swedish Alzheimer Foundation (#AF-742881), Hjärnfonden, Sweden (#FO2017-0243), the Swedish state under the agreement between the Swedish government and the County Councils, the ALF-agreement (#ALFGBG-715986), and the Alzheimer’s Association 2021 Zenith Award (ZEN-21-84849).

## Conflict of Interest

The authors declare that the research was conducted in the absence of any commercial or financial relationships that could be construed as a potential conflict of interest.

## Publisher’s Note

All claims expressed in this article are solely those of the authors and do not necessarily represent those of their affiliated organizations, or those of the publisher, the editors and the reviewers. Any product that may be evaluated in this article, or claim that may be made by its manufacturer, is not guaranteed or endorsed by the publisher.
